# Ambient synthesis of an iminium-linked covalent organic framework for synergetic RNA interference and metabolic therapy of fibrosarcoma†

**DOI:** 10.1039/d2sc02297d

**Published:** 2022-06-15

**Authors:** Le-Le Zhou, Qun Guan, Wei Zhou, Jing-Lan Kan, Yu-Bin Dong

**Affiliations:** College of Chemistry, Chemical Engineering and Materials Science, Collaborative Innovation Center of Functionalized Probes for Chemical Imaging in Universities of Shandong, Key Laboratory of Molecular and Nano Probes, Ministry of Education, Shandong Normal University Jinan 250014 China yubindong@sdnu.edu.cn; Department of Oncology, Shandong Provincial Hospital Affiliated to Shandong First Medical University Jinan 250021 China

## Abstract

Small interfering RNA (siRNA)-mediated gene silencing is a promising therapeutic approach. Herein, we report the ambient synthesis of a positively charged iminium-linked covalent organic framework by a three-component one-pot reaction. Through anion exchange and siRNA adsorption, the resulting multifunctional siRNA@ABMBP-COF, which possesses both the HK2 inhibitor 3-bromopyruvate and SLC7A11 siRNA, exhibits powerful synergistic antitumor activity against fibrosarcoma *via* the ferroptosis and apoptosis pathways.

## Abstract 2

An iminium-linked cationic covalent organic framework-based nanoagent that is synthesized under ambient conditions achieves synergistic RNA interference and metabolic therapy *via* effective siRNA delivery and timely lysosome escape.

## Graphical abstract

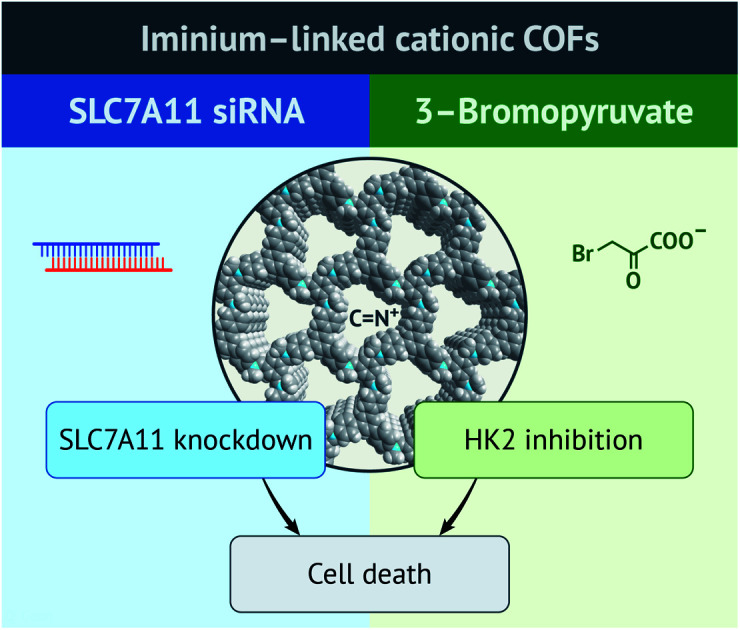

## Introduction

Small interfering RNAs (siRNAs) are powerful laboratory tools that can specifically inhibit targeted gene expression.^1,2^ The clinical translation of siRNA therapeutics is limited due to their negative charge, high molecular weight (approximately 14 kDa), ease of degradation, and low transmembrane uptake.^3^ Various delivery vectors, including viruses,^4^ proteins,^5^ liposomes,^6^ polymers,^7,8^ metal–organic frameworks,^9–11^ inorganic nanoparticles,^12^ and extracellular vesicles,^13^ have been developed to transport siRNAs into cells. However, the limited loading amount, insufficient lysosome escape, and difficulty in synergizing with other therapeutics greatly hinder their use in tumour treatment.^14,15^ Therefore, designing next-generation vectors for efficient siRNA delivery is urgent and important.

Since the pioneering work of Yaghi *et al.* in 2005,^16^ covalent organic frameworks (COFs), which are a class of porous materials, have shown great potential in drug delivery,^17–20^ protein encapsulation,^21–24^ phototherapy,^25–30^ and immunotherapy.^31–33^ In principle, COFs can adsorb nucleic acids to generate nucleic acid@COFs for oncotherapy. However, nucleic acid@COFs have never been used in antitumor treatments, which might also result from extremely low nucleic acid loading.^34–38^ We hypothesize that this bottleneck could be overcome by synthesizing positively charged COF-based carriers in which the loading amount of negatively charged therapeutic siRNA could be significantly improved *via* electrostatic interactions. Furthermore, the counterions in cationic COFs could be replaced with negatively charged metabolic inhibitors and chemotherapeutic drugs *via* ion exchange.^39^ Thus, multifunctional COF-based siRNA delivery and metabolic therapy could be logically achieved.

**Scheme 1:**
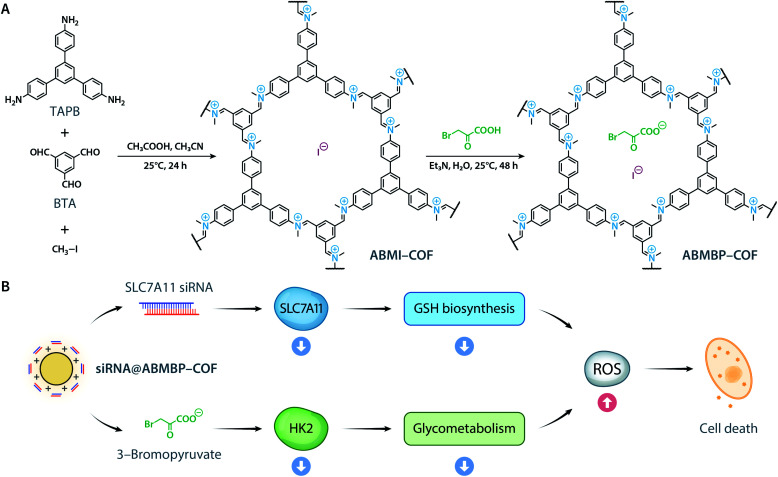
(A) Synthesis of ABMI-COF and ABMBP-COF. (B) ABMBP-COF with surface-adsorbed siRNA induced ferroptosis and apoptosis by inhibiting SLC7A11 expression and HK2 activity.

To date, the reported cationic COFs have been typically synthesized from positively charged monomers, including ethidium bromide,^40,41^ propidium iodide,^42^ imidazolium,^43–45^ quaternary ammonium,^46^ and guanidinium,^47^ under harsh solvothermal conditions. This energy-intensive and tedious approach is not conductive to large-scale synthesis. Herein, we report the ambient synthesis of the iminium-linked cationic ABMI-COF*via* a three-component one-pot reaction (Scheme 1A).^48–53^ Through ion exchange of the iodide counterion with 3-bromopyruvate, a hexokinase 2 (HK2) inhibitor,^54^ multifunctional ABMBP-COF was generated. Both ABMI-COF and ABMBP-COF possess high siRNA adsorption capacity (greater than 1 nmol mg^−1^) and can escape the lysosome. More importantly, after being loaded with solute carrier family 7 member 11 (SLC7A11) siRNA,^55^siRNA@ABMBP-COF could silence SLC7A11 and inhibit HK2, consequently achieving antitumor effects *in vitro* and *in vivo* through ferroptosis and apoptosis (Scheme 1B).

## Results and discussion

Inspired by the organic reaction reported by Raston *et al.*,^56^ the reaction of 1,3,5-tris(4-aminophenyl)benzene (TAPB), benzene-1,3,5-tricarbaldehyde (BTA), and iodomethane in CH_3_CN with acetic acid produced a 78% yield in ABMI-COF in after 24 h at room temperature (Fig. S1A†). Elemental analysis and inductively coupled plasma-mass spectrometry (ICP-MS) indicated that the obtained ABMI-COF had the molecular formula C_33_H_21_N_3_(CH_3_I)_2.80_, which is very close to the theoretical composition of C_33_H_21_N_3_(CH_3_I)_3_ (Fig. S1B†). Thermogravimetric analysis (TGA) showed that ABMI-COF was thermally stable up to approximately 380 °C (Fig. S1C†).

**Fig. 1:**
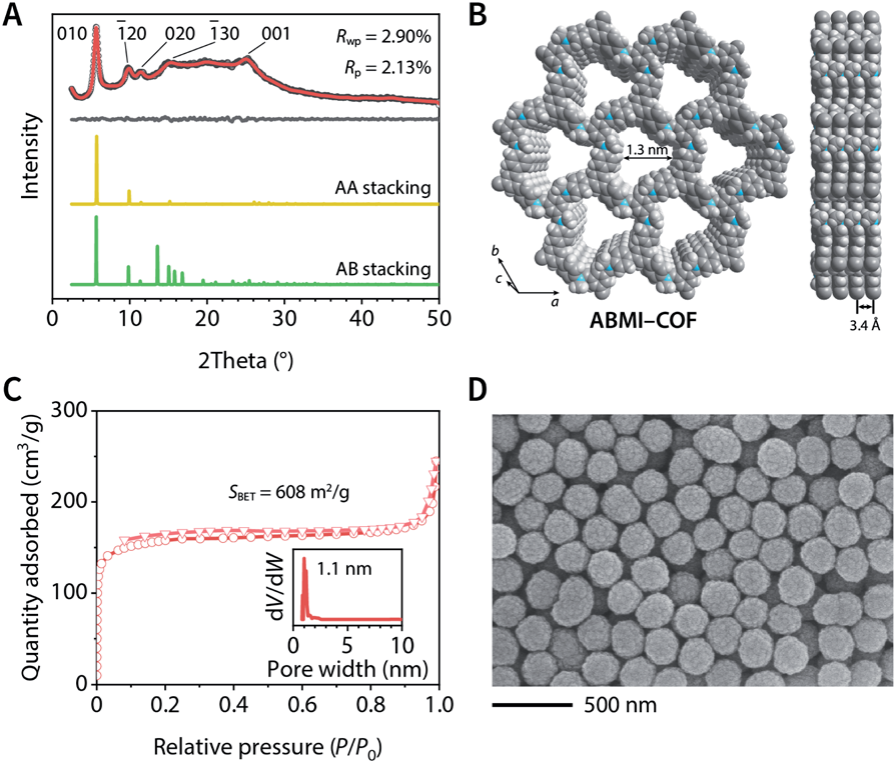
Characterization of ABMI-COF. (A) Experimental (black), Pawley-refined (red) and simulated (yellow and green) PXRD patterns and difference plot (grey). (B) Structural representations. (C) Nitrogen adsorption–desorption isotherm at 77 K and pore size distribution (inset). (D) SEM image.

The crystal structure of ABMI-COF was determined using Materials Studio software based on the measured powder X-ray diffraction (PXRD) pattern, in which a series of observed peaks at 2*θ* = 5.7°, 9.9°, 11.5°, 15.2°, and 26.1° were assigned to the (010), (−120), (020), (−130), and (001) facets, respectively (Fig. 1A). The results indicated that ABMI-COF possessed a 2D network with an eclipsed AA stacking mode (Fig. 1B). The Pawley refinement showed a negligible difference between the simulated and experimental PXRD patterns. ABMI-COF was assigned to the space group *P*3 with optimized parameters of *a* = *b* = 17.8 Å, *c* = 3.4 Å, *α* = *β* = 90°, *γ* = 120°, residuals *R*_wp_ = 2.90%, and *R*_p_ = 2.13% (Table S1†).

The type I N_2_ adsorption–desorption isotherm at 77 K of ABMI-COF showed that the Brunauer–Emmett–Teller (BET) surface area was *S*_BET_ = 608 m^2^ g^−1^ and the total pore volume at *P*/*P*_0_ = 0.99 was 0.38 cm^3^ g^−1^, confirming its porosity (Fig. 1C). The pore size distribution was determined by nonlocal density functional theory (NLDFT) analysis and indicated that it possessed a narrow pore diameter distribution centred at approximately 1.1 nm, which was consistent with the simulated structure.

The formation of ABMI-COF was also confirmed by spectroscopic methods. The Fourier transform infrared spectrum showed the characteristic peak of C=N^+^ at 1666 cm^−1^, and the appearance of peaks at 1248 and 1197 cm^−1^ were due to C–N^+^ (Fig. S1D†). The symmetrical and asymmetrical stretching vibrations of the CH_3_ group were located at 2872 and 2948 cm^−1^, respectively. Weak peaks of residual CHO and C=N were observed at 1697 and 1626 cm^−1^, respectively, indicating the presence of bonding defects.^57^ The observed carbon resonances in its ^13^C solid-state nuclear magnetic resonance spectrum showed that ABMI-COF contained iminium (182 ppm), methyl (53 ppm), and aromatic (100–150 ppm) species (Fig. S1E†).^58^ XPS analysis of ABMI-COF in the N1s region was deconvoluted into a C=N^+^ peak at 401.1 eV and a C=N peak at 398.3 eV (Fig. S1F†).^59^ Furthermore, two peaks with a well-separated spin–orbit component of 11.5 eV located at 619.2 and 630.7 eV were assigned to the iodide ion (Fig. S1G†).

Scanning electron microscopy (SEM) and transmission electron microscopy (TEM) images showed a uniform spherical morphology of ABMI-COF with a diameter of approximately 230 nm (Fig. 1D and S1H†). The dynamic light scattering (DLS) measurements showed a *z*-average size of 232.7 nm with a polydispersity index of 0.120 in phosphate-buffered saline, indicating its good dispersion (Fig. S1I†).

The iodide counterions within ABMI-COF could be partly exchanged by 3-bromopyruvate in a weakly basic triethylamine solution to generate ABMBP-COF, which had a molecular formula of C_33_H_21_N_3_(CH_3_)_2.80_I_0.48_(C_3_H_2_BrO_3_)_2.32_ based on ICP-MS and elemental analysis. As shown in Fig. S2,† ion exchange did not cause changes in crystallinity, structure, micromorphology, or dispersibility but resulted in a slight *S*_BET_ decrease (ABMBP-COF, 567 m^2^ g^−1^).

**Fig. 2:**
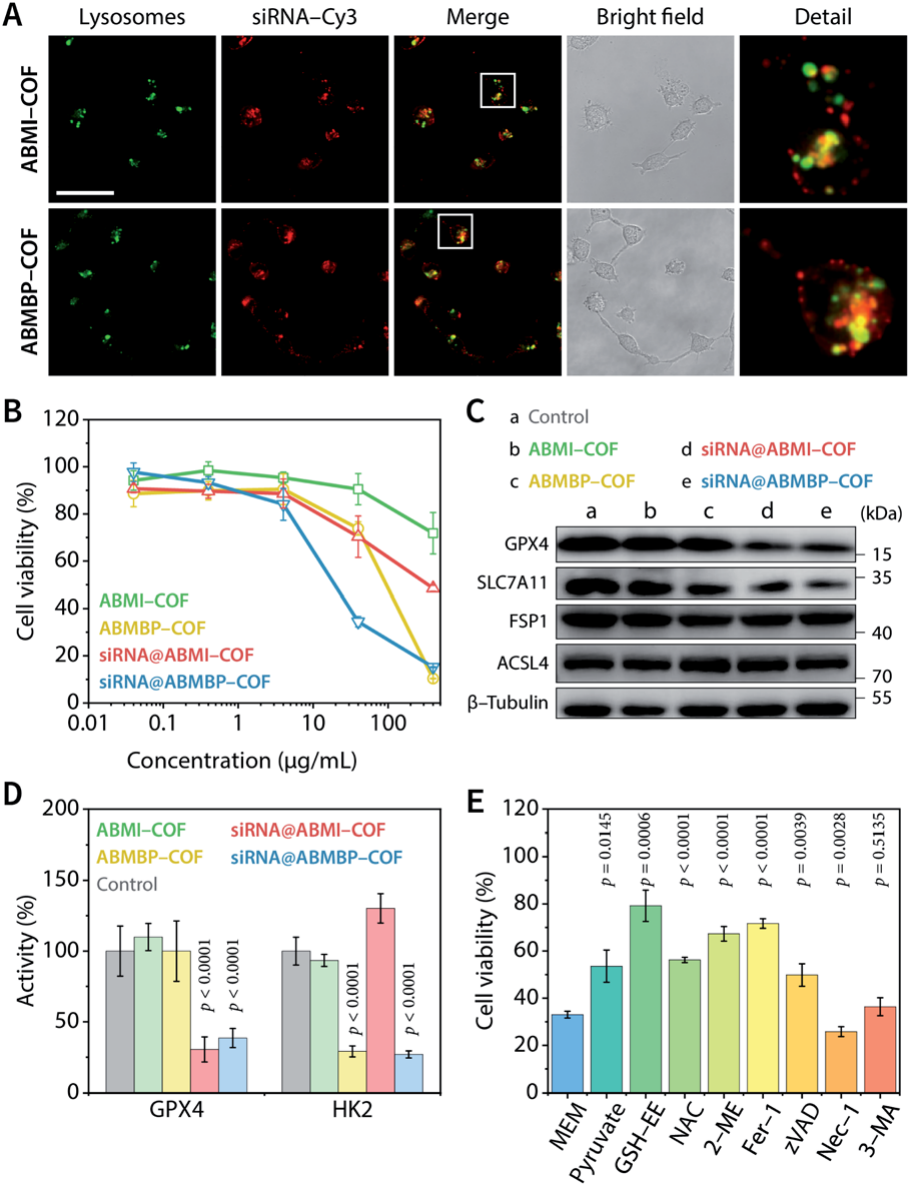
Ferroptosis- and apoptosis-related cell death. (A) Lysosome escape of siRNA-Cy3 in HT-1080 cells treated with siRNA-Cy3@ABMI-COF and siRNA-Cy3@ABMBP-COF. Scale bar, 50 μm. (B) Viability of HT-1080 cells based on the CCK-8 method. (C) Western blot analysis of GPX4, SLC7A11, FSP1, and ACSL4 in HT1080 cells. (D) GPX4 and HK2 enzymatic activity in HT-1080 cells. (E) Viability of HT-1080 cells treated with siRNA@ABMBP-COF (40 μg mL^−1^, COF equiv.) and cultured for an additional 48 h in the presence of sodium pyruvate, GSH-EE, NAC, 2-ME, Fer-1, zVAD, Nec-1, and 3-MA in minimum essential medium. The data are presented as the mean ± SD, *n* = 5 (B and E) or 4 (D), and were compared by one-way ANOVA followed by Dunnett's *post hoc* test (D) or Welch's ANOVA followed by Dunnett's T3 multiple comparison test (E).

Due to the iminium linkage, ABMI-COF and ABMBP-COF had positive zeta potentials of +28.2 and +25.0 mV, respectively, which endowed these nanoparticles with a high capacity to adsorb negatively charged siRNA. Not surprisingly, after adsorbing siRNA, the zeta potential of the nanoparticles decreased to approximately 60% of that before adsorption, while the hydrodynamic diameters based on DLS measurements were almost unchanged, indicating that siRNA adsorption did not lead to significant particle coagulation (Fig. S3A†). Furthermore, after adsorption, the fluorescence of surface-adsorbed Cy3-labelled siRNA (siRNA-Cy3) was effectively quenched *via* fluorescence resonance energy transfer caused by the spectral overlap between the Cy3 donor emission and COF acceptor absorption (Fig. S3B–D†). Fluorescent quantitative experiments showed that the saturated adsorption capacities of ABMI-COF and ABMBP-COF for siRNA were up to 1.2 and 1.1 nmol mg^−1^, respectively, which are significantly higher than those of electroneutral COFs (Fig. S3E†).^34–38,60^ Unsurprisingly, due to the cytomembrane affinity and proton sponge effect caused by the positive charges,^7,61^ the obtained siRNA@ABMI-COF and siRNA@ABMBP-COF readily entered HT-1080 cells within 4 h *via* pinocytosis (Fig. S4†) and then escaped from lysosomes into the cytoplasm (Fig. 2A). Their transfection efficiencies were superior to those of commercially available polyethylenimine and calcium phosphate and were comparable to those of Lipofectamine 2000 (Fig. S5†).

siRNA@ABMBP-COF, which contains the HK2 inhibitor 3-bromopyruvate, can cause oxidative stress and consequent cell death by inhibiting aerobic glycolysis and mitochondrial oxidative phosphorylation.^54^ Theoretically, the antitumor effect of 3-bromopyruvate could be further enhanced by blocking the biosynthesis of glutathione (GSH), which is the major intracellular response to oxidative stress.^62^ To examine this possibility, SLC7A11,^55,63^ a key transporter that is upstream of GSH biosynthesis, was selected as a therapeutic target. siRNA-mediated knockdown of SLC7A11 could inhibit cellular uptake of cystine, thereby blocking GSH synthesis and enhancing oxidative stress.^64^

According to CCK-8 cell viability assays, siRNA@ABMBP-COF (40 μg mL^−1^, COF equiv.) reduced HT-1080 cell viability to 34.5 ± 2.2% compared to the untreated group, which was significantly better than siRNA@ABMI-COF (70.3 ± 8.8%), ABMBP-COF (73.8 ± 2.8%), and ABMI-COF (90.5 ± 6.6%), suggesting a combined effect of SLC7A11 siRNA and 3-bromopyruvate (Fig. 2B). Clonogenic analysis was performed, and siRNA@ABMBP-COF-treated HT-1080 cells had the lowest clone formation compared with the other treatment groups, further supporting the obtained results (Fig. S6†).

The cell death mechanism induced by the cationic COF-based nanodrugs was investigated. After SLC7A11 siRNA was loaded, siRNA@ABMI-COF and siRNA@ABMBP-COF decreased SLC7A11 expression (Fig. 2C and S7†), which subsequently blocked GSH synthesis. As a result, a series of cellular biological changes were examined at 48 h, including decreases in the GSH concentration (Fig. S8A†), increases in cytoplasmic Fe^2+^ levels (Fig. S9†), reactive oxygen species (ROS) production (Fig. S10†) and lipid peroxidation (Fig. S11†), decreases in glutathione peroxidase 4 (GPX4) expression and activity (Fig. 2C and D), increased malonaldehyde concentrations (Fig. S8B†), and mitochondrial membrane potential loss (Fig. S12†). These results were consistent with the characteristics of ferroptosis.^65–67^ Furthermore, the expression of ferroptosis suppressor protein 1 (FSP1) and acyl-coenzyme A synthetase long-chain family member 4 (ACSL4) was intact (Fig. 2C and S7†), suggesting that HT-1080 cells triggered ferroptosis *via* the cyst(e)ine–GPX4–GSH axis.^68,69^

Notably, ABMBP-COF treatment contributed to GSH depletion (Fig. S8A†), ROS upregulation (Fig. S10†), and mitochondrial damage (Fig. S12†) but did not upregulate malonaldehyde content (Fig. S8B†) or downregulate GPX4 expression (Fig. 2C and S7†). These results suggested that 3-bromopyruvate induced cell death through an additional mechanism. After treatment with ABMBP-COF and siRNA@ABMBP-COF for 48 h, HK2 activity in HT-1080 cells decreased to less than 30% of that in the control group (Fig. 2D), and caspase 3 activation was detected by immunofluorescence staining (Fig. S13†), suggesting that the released 3-bromopyruvate induced apoptosis by triggering cellular energy stress. Interestingly, the combination of ABMBP-COF-induced energy stress and siRNA-induced ferroptosis was more effective in reducing GSH and elevating ROS than either treatment alone, emphasizing the distinct advantage of synergistic treatment (Fig. S8A and S10†).

Ferroptosis and apoptosis were further validated by cell rescue experiments in which different functional molecules were added to the media and cultured with siRNA@ABMBP-COF treated HT-1080 cells (Fig. 2E). Ferrostatin-1 (Fer-1)—a ferroptosis inhibitor—alleviated cell death, and direct supplementation with raw materials for GSH biosynthesis, such as glutathione ethyl ester (GSH-EE), *N*-acetyl-l-cysteine (NAC), and 2-mercaptoethanol (2-ME), restored cell viability to varying degrees,^70,71^ suggesting that GSH depletion promoted ferroptotic cell death. In addition, pyruvate, which is a final product of the glycolytic pathway, unblocked glucose metabolism and restored cell viability,^72^ and the apoptosis inhibitor Z-VAD-FMK (zVAD) inhibited cell death,^73^ suggesting that energy stress induced apoptosis. The necroptosis inhibitor necrostatin-1 (Nec-1) and the autophagy inhibitor 3-methyladenine (3-MA) had negligible effects on cell viability; thus, necroptosis- and autophagy-related cell death were excluded.^73^

**Fig. 3:**
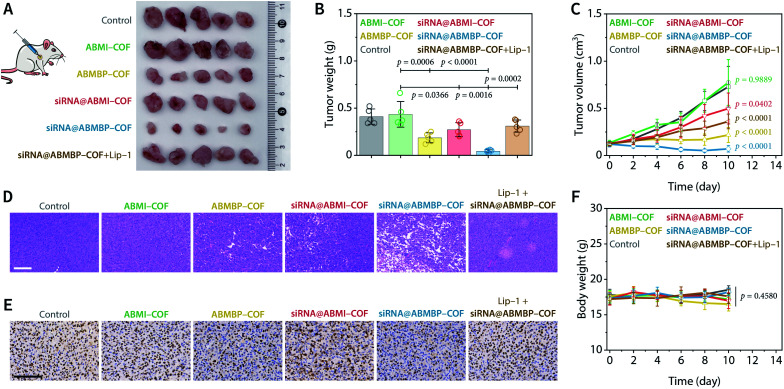
*In vivo* antitumor performance in HT-1080 tumour-bearing nude mice. (A) Photos of tumour tissue obtained by dissection at the end of treatment. (B) Weight of the obtained tumours. (C) Tumour growth curves. (D) H&E staining of the obtained tumours. Scale bar, 100 μm. (E) Ki67 immunohistochemical staining of the obtained tumours. Scale bar, 100 μm. (F) Body weight curves. Data are presented as the mean ± SD (*n* = 5) and compared by one-way (B) or two-way (C and F) ANOVA followed by Tukey's *post hoc* test.

Encouraged by the obtained results, *in vivo* antitumor activity was evaluated using an HT-1080 human fibrosarcoma xenograft model implanted in BALB/c nude mice. Tumours were collected on day 10 after intratumoral injection of the nanodrugs (0.8 mg mL^−1^, COF equiv.), and the results showed that the antitumor therapeutic efficacy was enhanced in the following order: ABMI-COF, siRNA@ABMI-COF, ABMBP-COF, and siRNA@ABMBP-COF (Fig. 3A and B). Specifically, siRNA@ABMBP-COF reduced the tumour volume to approximately 60% of that before treatment, while siRNA@ABMI-COF and ABMBP-COF exerted worse antitumoral effects, and ABMI-COF had almost no antitumor effect (Fig. 3C). Histopathological analysis of haematoxylin–eosin (H&E)-stained tumour tissues collected at the end of the treatment showed that the histological morphology of siRNA@ABMBP-COF-treated tumours was significantly different from that of the control group, as indicated by extensive cell membrane rupture, nuclear contraction, and loosely arranged cells, indicating cellular damage (Fig. 3D). Ki67 is a nuclear antigen associated with cell proliferation and cancer prognosis and is a cellular marker for measuring the proliferative potential of cancer cells.^74^ The immunohistochemical staining results (Fig. 3E) showed that siRNA@ABMBP-COF resulted in a lower ratio of Ki67-positive cells than ABMBP-COF and siRNA@ABMI-COF, indicating the suppression of tumour proliferation. These experimental results are consistent with the trend in the tumour growth curve.

The ferroptosis inhibitor liproxstatin-1 (Lip-1) clearly counteracted the tumour treatment effect induced by siRNA@ABMBP-COF (Fig. 3A–E) and the terminal deoxynucleotidyl transferase-mediated deoxyuridine triphosphate nick-end labelling (TUNEL) assay demonstrated slight increases in the proportion of apoptotic cells in the groups of ABMBP-COF and siRNA@ABMBP-COF (Fig. S14†), suggesting ferroptosis- and apoptosis-related antitumor mechanism *in vivo*. Furthermore, the levels of ferroptosis- and apoptosis-related metabolites and enzymatic activities were determined on day 4. Compared with monotherapy with ABMBP-COF and siRNA@ABMI-COF, the siRNA@ABMBP-COF-induced combination treatment resulted in significant intratumoral GSH downregulation and malonaldehyde upregulation, suggesting the presence of oxidative stress and ferroptosis *in vivo* (Fig. S15A and B†). Additionally, siRNA@ABMBP-COF inhibited intratumoral HK2 activity, but there was no significant difference in GPX4 activity (Fig. S15C and D†), suggesting the presence of a compensatory mechanism in solid tumours,^75^ which was different from the *in vitro* observations. Although siRNA@ABMBP-COF achieved an obvious antitumor effect, it is clear that the GPX4-related compensatory mechanism *in vivo* is unfavourable for tumour therapy. We believe that antitumor therapy can be further optimized by combining GPX4 inhibitors^76^ or radiotherapy,^77,78^ which will be investigated in the future.

The systemic toxicity of the nanodrugs to nude mice during the treatment was negligible, which was confirmed by a lack of significant weight loss in mice during the treatment (Fig. 3F) and H&E staining of major organs collected at the end of treatment (Fig. S16A†). Routine blood and biochemical examinations showed negligible adverse effects of the nanodrugs on liver function, kidney function, and the blood system in healthy nude mice (Fig. S16B and C†). Therefore, the nanodrugs have no obvious acute toxicity and have acceptable biosafety.

## Conclusions

In conclusion, we reported the synthesis of an iminium-linked positively charged COF by a three-component one-pot reaction under ambient conditions. Through anion exchange and siRNA adsorption, the resulting multifunctional COF-based nanodrug exerts potent combined antitumor activity against HT-1080 tumour cells and tissues *via* ferroptosis and apoptosis. This study not only enriches COF synthetic methodology but also highlights cationic COF as a promising platform for siRNA-mediated combination therapy.

## Experimental section

### Synthesis of ABMI-COF

The mixture of TAPB (562.3 mg, 1.6 mmol), BTA (259.4 mg, 1.6 mmol), acetonitrile (200 mL), acetic acid (32 mL), and iodomethane (32 mL) was stirred at 800 rpm and 25 °C for 24 h. The precipitate was collected by centrifugation at 12 000 rpm (14 800 × *g*) for 30 min at 4 °C and washed 3 times with acetonitrile and then 3 times with ethanol. Finally, the precipitate was dried under supercritical CO_2_ to obtain ABMI-COF as an orange-red powder. The yield was 1.1 g (78%).

### Synthesis of ABMBP-COF

ABMI-COF (50 mg) was dispersed in an aqueous solution (100 mL) containing 3-bromopyruvic acid (83.5 mg, 0.5 mmol) and triethylamine (100 μL, 0.7 mmol). The mixture was stirred at 600 rpm and 25 °C for 12 h. The precipitate was separated by centrifugation at 12 000 rpm (14 800 × *g*) for 30 min at 4 °C. The dispersion–stirring–centrifugation process was repeated 4 times. The obtained precipitate was washed three times with water and once with ethanol and was dried under vacuum to obtain ABMBP-COF as an orange-red powder. The yield was 50 mg.

### Animal experimentation

All animal procedures were reviewed and approved by the Ethics Committee of Shandong Normal University (Jinan, China; application number AEECSDNU2021009). Further information regarding experimental procedures are stated in the ESI.†

## Author contributions

Conceptualization, Y.-B. Dong. Investigation, L.-L. Zhou, Q. Guan, W. Zhou, and J.-L. Kan. Methodology, L.-L. Zhou, Q. Guan, and Y.-B. Dong. Project administration, Y.-B. Dong. Resources, Y.-B. Dong. Supervision, Y.-B. Dong. Visualization, Q. Guan. Writing – original draft, L.-L. Zhou and Q. Guan. Writing – review & editing, L.-L. Zhou, Q. Guan, and Y.-B. Dong.

## Data availability

The data that support the findings of this study are presented in the paper and the ESI.†

## Conflicts of interest

There are no conflicts to declare.

## Supplementary Material

SC-013-D2SC02297D-s001
