# Supplementary material for: Ambient synthesis of an iminium-linked covalent organic framework for synergetic RNA interference and metabolic therapy of fibrosarcoma
Source: Chem Sci. 2022 Jun 15;13(26):7846–54. doi: 10.1039/d2sc02297d (PMC9258322; doi:10.1039/d2sc02297d)
Supplement: SC-013-D2SC02297D-s001 [file SC-013-D2SC02297D-s001.pdf]

Electronic Supplementary Material (ESI) for Chemical Science.
This journal is © The Royal Society of Chemistry 2022

Chem. Sci., 2022, DOI: 10.1039/D2SC02297D

Electronic supplementary information

Ambient synthesis of an iminium-linked covalent organic framework for synergetic RNA interference and metabolic therapy of fibrosarcoma

Le-Le Zhou,[a,‡] Qun Guan,[a,‡] Wei Zhou,[b] Jing-Lan Kan,[a] and Yu-Bin Dong[a,*]

a. College of Chemistry, Chemical Engineering and Materials Science, Collaborative Innovation Center of Functionalized Probes for Chemical Imaging in Universities of Shandong, Key Laboratory of Molecular and Nano Probes, Ministry of Education, Shandong Normal University, Jinan 250014, China. *E-mail: yubindong@sdnu.edu.cn (Y.-B. Dong)

b. Department of Oncology, Shandong Provincial Hospital Affiliated to Shandong First Medical University, Jinan 250021, China.

‡ These authors contributed equally.

1. Experimental materials S2
2. Experimental instrumentation S3
3. Characterization of **ABMI-COF** S4
4. Characterization of **ABMBP-COF** S6
5. siRNA adsorption and fluorescence quenching effects S6
6. Cell culture S7
7. Cellular uptake and lysosome escape behaviours S7
8. Transfection efficiency S8
9. Cell viability assays S9
10. Clonogenic assays S9
11. Western blotting S10
12. Enzymatic activity assays S10
13. Intracellular GSH and malondialdehyde measurement S10
14. Intracellular Fe^{2+} analysis S11
15. Intracellular ROS measurement S11
16. Intracellular lipid peroxide measurement S12
17. Mitochondrial membrane potential measurement S12
18. Immunofluorescence imaging of active caspase 3 .... S13
19. Cell death rescue experiments S14
20. Experimental animals S14
21. *In vivo* antitumor efficacy S14

1. Experimental materials

1,3,5-Tris(4-aminophenyl)benzene was purchased from Kylpharm (Shanghai, China).

Benzene-1,3,5-tricarbaldehyde was purchased from Jilin Chinese Academy of Sciences – Yanshen Technology (Changchun, China).

Acetonitrile, ethanol, methanol, triethylamine, and acetic acid were purchased from Sinopharm (Beijing, China).

3-Bromopyruvic acid was purchased from Macklin (Shanghai, China).

Iodomethane was purchased from Xiya Reagent (Linyi, China).

Ferrostatin-1, liproxstatin-1, necrostatin-1, deferoxamine mesylate (DFOM), propidium iodide, 2',7'-dichlorodihydrofluorescein diacetate (DCFH-DA), 5,5',6,6'-tetrachloro-1,1',3,3'-tetraethylbenzimidazolocarbocyanine iodide (JC-1), the protease inhibitor cocktail (Cat# HY-K0010), and the phosphatase inhibitor cocktail (Cat# HY-K0022) were purchased from MedChemExpress (Shanghai, China).

The CCK-8 assay kit and FerroOrange were purchased from Dojindo (Shanghai, China).

Minimum essential medium (MEM), Opti-MEM I reduced serum medium (Opti-MEM), Hank's balanced salt solution (HBSS), sodium pyruvate (100 mM), GlutaMAX (100×), BODIPY 581/591 C11 (C_{11}-BODIPY, Cat# D3861), Alexa Fluor 488 cadaverine (Cat# A30676), SYTO 9 Green (Cat# S34854), LysoTracker Green DND-26 (Cat# L7526), Lipofectamine 2000 transfection reagent (Cat# 11668500), RIPA lysis and extraction buffer (Cat# 89900), the BCA protein assay kit (Cat# 23227), Alexa Fluor 488-conjugated goat anti-rabbit IgG (H+L) secondary antibody (Cat# A-11008), rabbit anti-active caspase 3 (Cat# MA5-32015), and the PageRuler prestained protein ladder (10–180 kDa, Cat# 26616) were purchased from Thermo Fisher Scientific Inc.

SDS–PAGE sample loading buffer (Cat# P0015) and the calcium phosphate cell transfection kit (Cat# C0508) were purchased from Beyotime (Shanghai, China).

3-Methyladenine, *N*-acetyl-*L*-cysteine, 2-mercaptoethanol, Tween-20, and Triton X-100 were purchased from TCI (Shanghai) Development Co., Ltd.

Glutathione ethyl ester was purchased from Sigma–Aldrich (Shanghai, China).

A reduced glutathione assay kit and haematoxylin–eosin (H&E) stain kit were purchased from Nanjing Jiancheng Bioengineering Institute (China).

A hexokinase II (HK2) inhibitor assay kit, glutathione peroxidase 4 (GPX4) assay kit, malonaldehyde assay kit, rabbit anti-ACSL4 (Cat# ab155282), and rabbit anti-GPX4 (Cat# ab125066) were purchased from Abcam (Shanghai, China).

A TUNEL apoptosis detection kit (Alexa Fluor 640), polyethylenimine 25000 (PEI 25k), and polyethylenimine 40000 (PEI 40k) were purchased from Yeasen Biotechnology (Shanghai) Co., Ltd.

SLC7A11 siRNA (Cat# hs.Ri.SLC7A11.13) and Cy3-labelled control siRNA-Cy3 (Cat# 51-01-03-08) were purchased from Integrated DNA Technologies, Inc.

Rabbit anti-FSP1 (Cat# 20886-1-AP) and horseradish peroxidase (HRP)-conjugated goat anti-rabbit IgG (H+L) secondary antibody (Cat# SA00001-2) were purchased from Proteintech (Wuhan, China).

Z-VAD-FMK was purchased from Selleck Chemicals LLC.

Rabbit anti-SLC7A11 (Cat# 12691) and rabbit anti-Ki67 (Cat# 9027) were purchased from Cell Signaling Technology, Inc.

Rabbit anti-β-tubulin (Cat# ET1602-4) was purchased from HuaBio (Hangzhou, China).

A chemiluminescence detection kit (Cat# E412-01) was purchased from Vazyme (Nanjing, China).

Paraformaldehyde fixation solution was purchased from Babio (Jinan, China).

Giemsa staining solution and goat serum were purchased from Beijing Solarbio Science & Technology Co., Ltd.

Tris-buffered saline powder (Cat# G0001-2L), SDS–PAGE running buffer powder (Cat# G2018-1L), and PAGE transfer buffer powder (Cat# G2017-1L) were purchased from Servicebio (Wuhan, China).

Trypsin (0.25 wt%) and ethylenediaminetetraacetic acid (EDTA, 0.02 wt%) in Puck's saline A (trypsin/EDTA solution) was purchased from Biological Industries (Sartorius Group, USA).

Certified foetal bovine serum (FBS) and phosphate-buffered saline (PBS) were purchased from VivaCell (Shanghai, China).

Normocin was purchased from Invivogen (USA).

2. Experimental instrumentation

Supercritical carbon dioxide drying was performed using a Tousimis Samdri-PVT-3D critical point dryer. Damp solids were contained in folded filter paper secured with a staple and dried with a 15 min purge time and 15 min equilibration time after heating.

Powder X-ray diffraction (PXRD) patterns were obtained on a SmartLab SE X-ray powder diffractometer (Rigaku, Japan) with Cu $K\alpha$ line focused radiation (λ = 1.54056 Å) in the range of 2θ = 2.50°–50.00° at a step size of 0.01°.

Nitrogen-adsorption isotherms of samples that were degassed under vacuum at 120°C for 8 h were measured at 77 K using a Micromeritics ASAP2020 HD88 surface area and porosity analyser. The Brunauer–Emmett–Teller (BET) equation was used to calculate the specific surface areas.

Fourier transform infrared (FT-IR) spectra were obtained on a Thermo Scientific Nicolet iS50 FT-IR spectrometer equipped with a diamond attenuated total reflection (ATR) module between 4000–400 cm^{-1}. Each spectrum represented an average of 16 scans.

Thermogravimetric analysis (TGA) was performed with a Mettler Toledo TGA/DSC3+ thermogravimetric analyser. Approximately 5.0 mg of dried sample was analysed from room temperature to 900°C at a heating rate of 10°C/min under a N_2 atmosphere.

^{13}C Cross-polarization/magic-angle spinning solid-state NMR (^{13}C CP/MAS ssNMR) spectra were recorded on a Bruker Advance III 400 MHz NMR spectrometer.

Ultraviolet–visible (UV–vis) absorption spectra were recorded on a Shimadzu UV-2700 double-beam UV–vis spectrophotometer using a 10 mm quartz cuvette.

Inductively coupled plasma–mass spectrometry (ICP–MS) measurements were carried out using a PerkinElmer NexION 300X ICP–MS.

The hydrodynamic particle size and zeta potential were measured using a Malvern Zetasizer Nano ZS90 system.

Transmission electron microscopy (TEM) images were recorded on a Hitachi HT7700 120 kV compact-digital instrument. TEM samples were prepared in methanol by sonication for 5 min, followed by application on a carbon-coated copper TEM grid (200 mesh, Beijing Zhongxing Bairui Technology Co., Ltd.), and air drying at room temperature.

Scanning electron microscopy (SEM) images were recorded using a Hitachi SU8010 instrument. SEM samples were prepared by depositing a diluted suspension onto silicon wafers (approximately 3 × 3 mm, Beijing Zhongxing Bairui Technology Co., Ltd.), followed by air drying and coating with a thin layer of Pt to increase the contrast.

Microplate assays were conducted using a Molecular Devices SpectraMax i3x microplate detection system.

Western blot images were obtained using a GE Healthcare Amersham Imager 600 luminescent image analyser.

Laser scanning confocal fluorescence images of cells were captured with a Leica TCS SP8 confocal laser scanning microscope equipped with 405 nm, 458 nm, 488 nm, 514 nm, 561 nm, and 633 nm lasers. The imaging scan speed was 400 Hz, and transmitted light was used to find the areas of interest to reduce photodamage to the sample. Glass bottom dishes and 4/8-well chamber slides (Cellvis, USA) were used for cell culture in each experiment. The original culture media and PBS were replaced with HBSS supplemented with HEPES (15 mM, pH 7.4) and GlutaMAX (1×) to provide better buffering capacity under normal CO_2 concentrations before live cell imaging.

Flow cytometric analysis was performed using a BD FACSCalibur flow cytometer.

3. Characterization of ABMI-COF

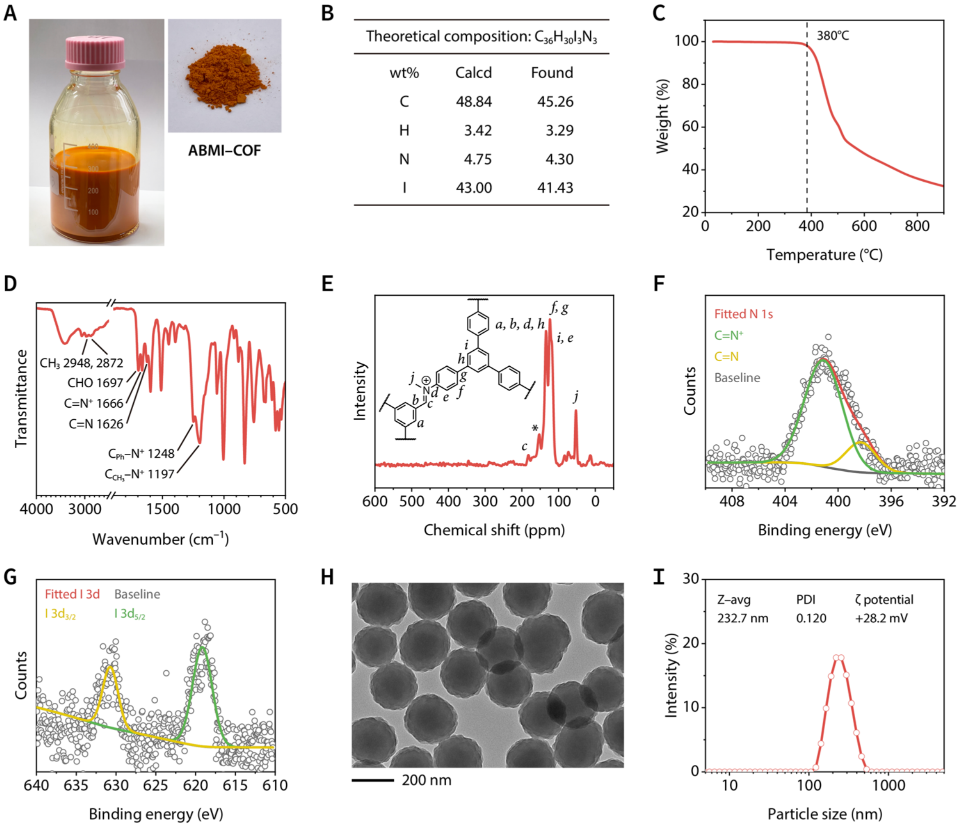

Fig. S1 Characterization of **ABMI-COF**. (A) Photographs of the synthesis system and obtained **ABMI-COF** powder. (B) Elemental compositions. C, N, and H contents were determined by elemental analysis, and the content of I was determined by ICP–MS. (C) TGA curve. (D) ATR FT-IR spectrum. (E) ^{13}C CP/MAS ssNMR spectrum. *Residual imine C. (F, G) High-resolution XPS spectra in the N 1s (F) and I 3d (G) regions. (H) TEM image. (I) DLS particle size distribution plot.

Table S1 Atomic coordinates of the AA- and AB-stacking modes of **ABMI-COF**.

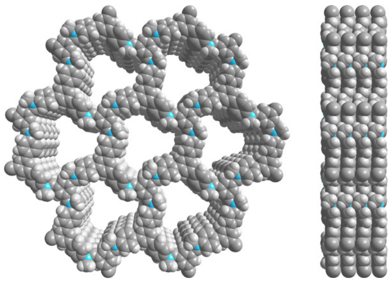

AA-stacking mode

Space group: $P3$ (143)

$a = b = 17.8212$ Å, $c = 3.4124$ Å

$\alpha = \beta = 90°$, $\gamma = 120°$

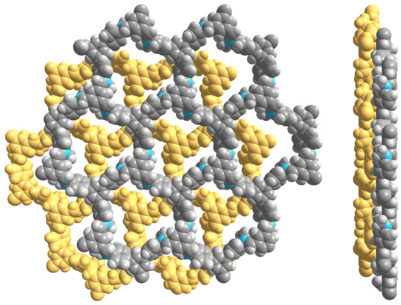

AB-stacking mode

Space group: $P6_3$ (173)

$a = b = 17.9821$ Å, $c = 7.8753$ Å

$\alpha = \beta = 90°$, $\gamma = 120°$

	x	*y*	*z*		*x*	*y*	*z*
C1	2.35603	−0.24699	−0.67681	C1	2.35969	−0.24619	−0.91469
C2	2.26907	−0.31068	−0.67333	C2	2.27251	−0.30709	−0.91370
C3	2.20699	−0.28051	−0.71212	C3	2.20975	−0.27837	−0.91979
C4	1.93490	−0.08579	−0.67406	C4	1.93859	−0.08787	−0.75308
C5	2.02059	−0.06600	−0.66318	C5	2.02730	−0.06219	−0.74864
C6	2.04252	−0.13456	−0.60546	C6	2.05564	−0.12792	−0.75976
C7	1.98767	−0.22032	−0.72560	C7	2.02993	−0.18392	−0.89218
C8	2.01311	−0.28186	−0.68526	C8	2.05118	−0.24906	−0.90916
C9	2.09478	−0.25755	−0.53921	C9	2.11143	−0.24886	−0.79338
C10	2.14697	−0.17344	−0.40310	C10	2.14636	−0.18549	−0.67279
C11	2.12052	−0.11373	−0.42714	C11	2.11664	−0.12981	−0.65321
N12	2.12978	−0.31397	−0.56272	N12	2.14832	−0.30221	−0.80795
C13	2.07976	−0.40140	−0.40723	C13	2.13619	−0.35807	−0.66229
H14	2.37214	−0.18079	−0.70384	H14	2.38010	−0.17842	−0.91957
H15	2.23559	−0.21666	−0.83334	H15	2.22382	−0.22621	−1.00790
H16	1.88469	−0.15230	−0.67648	H16	1.89104	−0.15531	−0.76216
H17	1.92740	−0.23953	−0.86899	H17	2.01064	−0.15861	−0.99104
H18	1.97185	−0.34643	−0.79160	H18	2.02518	−0.29467	−1.01164
H19	2.21054	−0.15231	−0.29185	H19	2.19327	−0.18069	−0.58082
H20	2.16237	−0.05115	−0.30364	H20	2.12758	−0.10664	−0.53417
H21	2.11953	−0.41523	−0.21275	H21	2.19814	−0.34031	−0.59823
H22	2.05423	−0.44994	−0.63738	H22	2.10479	−0.42489	−0.70657
H23	2.02318	−0.40914	−0.24551	H23	2.09367	−0.35408	−0.56327

4. Characterization of ABMBP-COF

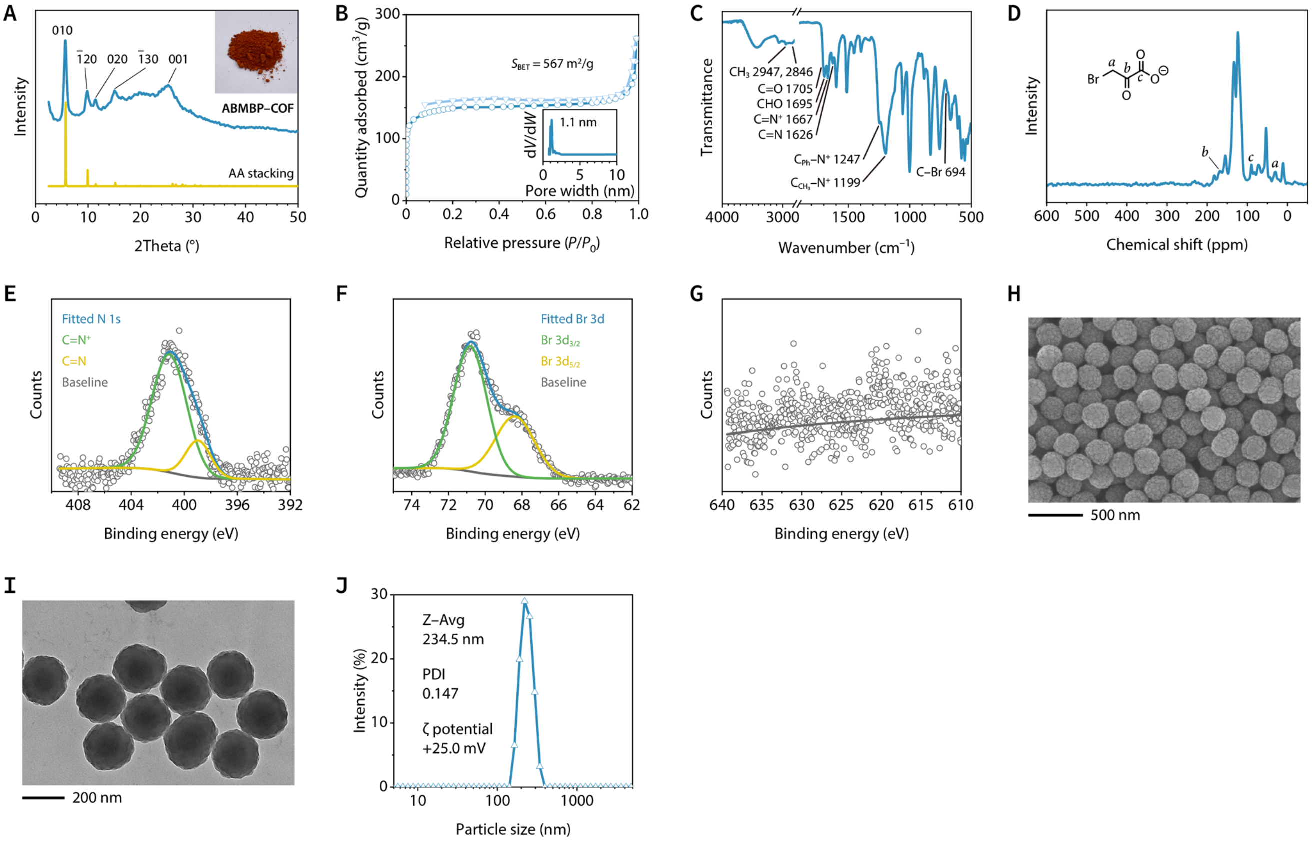

Fig. S2 Characterization of **ABMBP-COF**. (A) PXRD pattern. Inset: a photo of the **ABMBP-COF** powder. (B) N_2 adsorption and desorption isotherms at 77 K. Inset: pore width distribution plot based on the NLDFT model. (C) ATR FT-IR spectrum. (D) ^{13}C CP/MAS ssNMR spectrum. (E–G) High-resolution XPS spectra in the N 1s (E), Br 3d (F), and I 3d (G) regions. (H) SEM image. (I) TEM image. (J) DLS particle size distribution plot.

5. siRNA adsorption and fluorescence quenching effects

ABMI-COF and **ABMBP-COF** (1.0 mg/mL, 100 μL) were mixed with siRNA-Cy3 (0.5 μM, 100 μL) and shaken at room temperature for 10 min. Subsequently, the mixtures were diluted to 2.0 mL with PBS for DLS and zeta potential measurements.

In a black 96-well plate, **ABMI-COF** and **ABMBP-COF** (0–200 μg/mL, 100 μL) were mixed with siRNA-Cy3 (50 nM, 100 μL). The 96-well plate was shaken at room temperature for 10 min. Subsequently, the fluorescence intensity at 564 nm after excitation at 514 nm was measured using a multimode microplate detection system. The concentration of added **ABMI-COF** and **ABMBP-COF** was used as the horizontal axis, and the fluorescence intensity was used as the vertical axis to plot the fluorescence quenching curves.

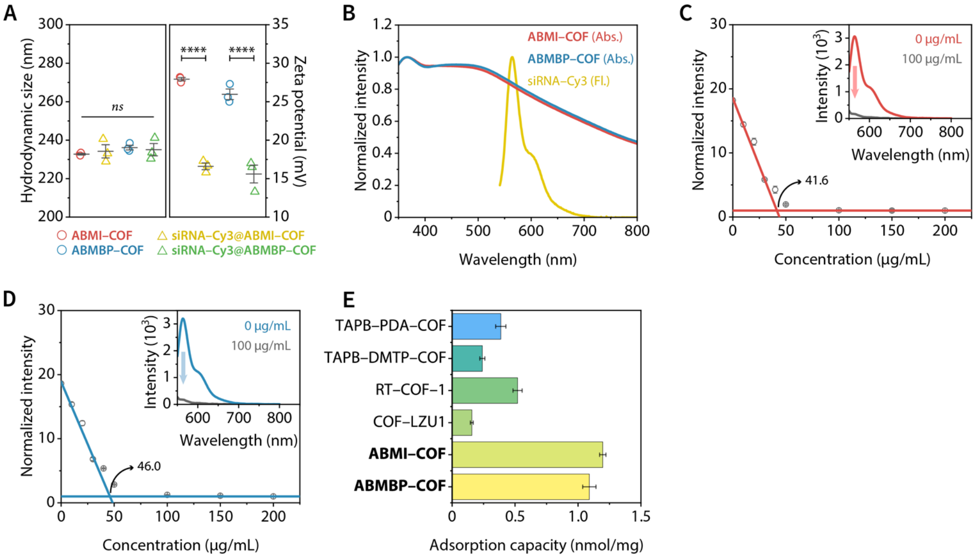

Fig. S3 siRNA adsorption and fluorescence quenching effects. (A) Changes in hydrodynamic size and zeta potential due to siRNA adsorption. The data are presented as the mean ± SD ($n = 3$) and were compared by one-way ANOVA followed by Tukey's post-hoc test. ****$p < 0.0001$, *ns*, no significance ($p > 0.05$). (B) Fluorescence spectrum of siRNA-Cy3 (excitation wavelength of 514 nm) and UV–vis spectra of **ABMI-COF** and **ABMBP-COF** in PBS. (C–D) Cy3 fluorescence quenching due to adsorption of siRNA-Cy3 on **ABMI-COF** (C) and **ABMBP-COF** (D). Inset: fluorescence spectra of Cy3 at different concentrations of **ABMI-COF** and **ABMBP-COF**. (E) Saturated adsorption capacity of COFs for siRNA-Cy3.

6. Cell culture

The HT-1080 (human fibrosarcoma) cell line was provided by the Cell Bank, Chinese Academy of Sciences (Shanghai, China). HT-1080 cells were cultured in MEM supplemented with FBS (10 vol%) and normocin (100 μg/mL) at 37°C in a water-jacketed CO_2 incubator with CO_2 (5 vol%).

7. Cellular uptake and lysosome escape behaviours

HT-1080 cells were treated with Alexa Fluor 488-labelled **ABMI-COF** and **ABMBP-COF** (1.0 mL, 40 μg/mL, COF equiv.) for 4 h in a CO_2 incubator and washed twice with PBS. After an additional 4 h of incubation, the cells were collected using a trypsin/EDTA solution and analysed by flow cytometry. Untreated cells were used as the control group.

siRNA-Cy3 (10 μM, 8 μL) was mixed with **ABMI-COF** and **ABMBP-COF** (800 μg/mL, 100 μL), and the mixture was shaken for 10 min at room temperature to obtain **siRNA-Cy3@ABMI-COF** and **siRNA-Cy3@ABMBP-COF**.

HT-1080 cells were treated with Alexa Fluor 488-labelled **siRNA-Cy3@ABMI-COF** and **siRNA-Cy3@ABMBP-COF** (100 μL, 40 μg/mL, COF equiv.) for 4 h in a CO_2 incubator and washed twice with PBS. After an additional 4 h of incubation, laser scanning confocal fluorescence images were captured. The green signal of Alexa Fluor 488 was recorded with an excitation wavelength of 488 nm and an emission wavelength range of 500–540 nm. The red signal of Cy3 was recorded at an excitation wavelength of 514 nm and an emission wavelength range of 545–585 nm.

HT-1080 cells were treated with **siRNA-Cy3@ABMI-COF** and **siRNA-Cy3@ABMBP-COF** (100 μL, 40 μg/mL, COF equiv.) for 4 h in a CO_2 incubator and washed twice with PBS. After an additional 1–4 h of incubation, the cells were incubated with LysoTracker Green DND-26 (200 μL, 100 nM) for 10 min and washed twice with PBS. Then, laser scanning confocal fluorescence images were captured. The green signal of LysoTracker Green DND-26 was recorded with an excitation wavelength of 488 nm and an emission wavelength

range of 500–540 nm. The red signal of Cy3 was recorded at an excitation wavelength of 514 nm and an emission wavelength range of 545–585 nm.

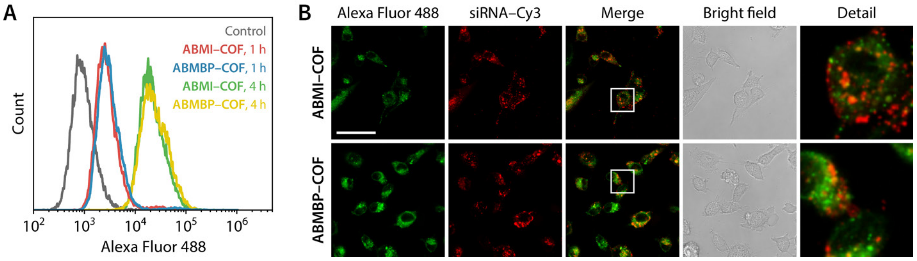

Fig. S4 Cellular uptake and siRNA-Cy3 release. (A) Flow cytometric analysis of the cellular uptake of Alexa Fluor 488-labelled **ABMI-COF** and **ABMBP-COF** (40 μg/mL, COF equiv.) by HT-1080 cells. (B) Desorption of siRNA-Cy3 from Alexa Fluor 488-labelled **ABMI-COF** and **ABMBP-COF** in HT-1080 cells treated with Alexa Fluor 488-labelled **siRNA-Cy3@ABMI-COF** and **siRNA-Cy3@ABMBP-COF** (40 μg/mL, COF equiv.). Scale bar, 50 μm.

8. Transfection efficiency

Transfection complexes with a final siRNA-Cy3 concentration of 100 nM were prepared as follows: (i) in the Opti-MEM group, siRNA-Cy3 (10 μM, 1 μL) was added to Opti-MEM (100 μL); (ii) in the **ABMI-COF** group, siRNA-Cy3 (10 μM, 1 μL) was mixed with **ABMI-COF** (100 μg/mL, 100 μL) and shaken for 10 min at room temperature to obtain the transfection complex; (iii) in the **ABMBP-COF** group, siRNA-Cy3 (10 μM, 1 μL) was mixed with **ABMBP-COF** (100 μg/mL, 100 μL) and shaken for 10 min at room temperature to obtain the transfection complex; (iv) in the Lipo2000 group, siRNA-Cy3 (10 μM, 1 μL) was mixed with Opti-MEM (50 μL) to obtain solution A, Lipofectamine 2000 (0.5 μL) was mixed with Opti-MEM (50 μL) to obtain solution B, and after 5 min of incubation, solution A and solution B were mixed and incubated at room temperature for 10 min to obtain the transfection complex; (v) in the PEI 25k group, siRNA-Cy3 (10 μM, 1 μL) was diluted in Opti-MEM (100 μL), mixed with PEI 25k (0.5 μL, 0.5 mg/mL) and incubated at room temperature for 15 min to obtain the transfection complex; (vi) in the PEI 40k group, siRNA-Cy3 (10 μM, 1 μL) was diluted in Opti-MEM (100 μL), mixed with PEI 40k (0.5 μL, 0.5 mg/mL) and incubated at room temperature for 15 min to obtain the transfection complex; and (vii) in the $Ca_3(PO_4)_2$ group, according to the guidelines of the calcium phosphate cell transfection kit, siRNA-Cy3 (10 μM, 1 μL) was diluted in $CaCl_2$ solution (50 μL), and the dilution was mixed with BBS solution (50 μL) and incubated at room temperature for 15 min to obtain the transfection complex.

For the transfection experiment, HT-1080 cells were cultured in 8-well chamber slides and washed twice with PBS, and MEM (60 μL) was added to each chamber. After 1 h of incubation, the freshly prepared transfection complex (40 μL) was added to each chamber. After 4 h of incubation, the cells were washed twice with PBS. After an additional 4 h of incubation, the cells were incubated with SYTO 9 Green (200 μL, 5.0 μM) for 30 min and washed twice with PBS. Then, laser scanning confocal fluorescence images were captured. The green images of SYTO 9 Green were captured with an excitation wavelength of 488 nm and an emission wavelength range of 500–540 nm. The red images of Cy3 were captured at an excitation wavelength of 514 nm and an emission wavelength range of 545–585 nm.

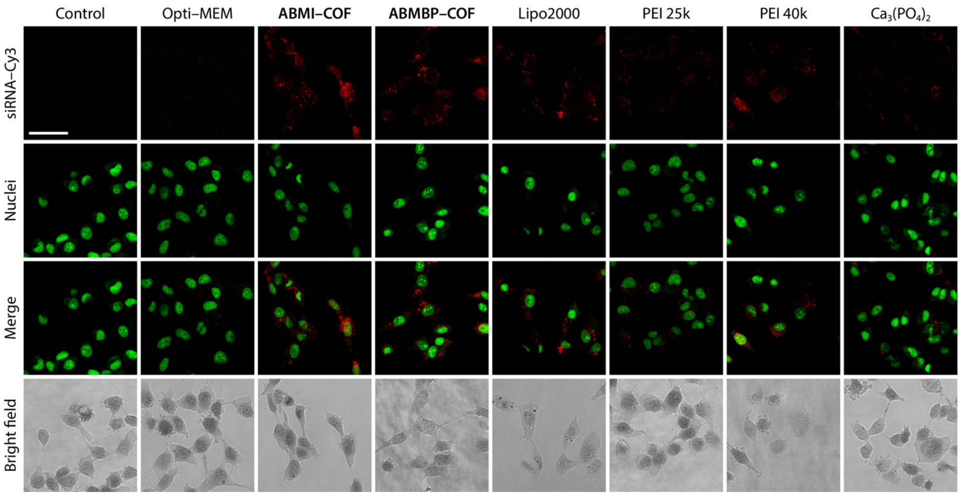

Fig. S5 Comparisons of transfection efficiencies. Scale bar, 50 μm.

9. Cell viability assays

SLC7A11 siRNA (10 μM, 8 μL) was mixed with **ABMI-COF** and **ABMBP-COF** (800 μg/mL, 100 μL), and the mixture was shaken for 10 min at room temperature to obtain **siRNA@ABMI-COF** and **siRNA@ABMBP-COF**.

HT-1080 cells cultured in 96-well plates were treated with **ABMI-COF**, **ABMBP-COF**, **siRNA@ABMI-COF**, and **siRNA@ABMBP-COF** (100 μL, 0–400 μg/mL, COF equiv.) for 4 h in a CO_2 incubator and washed twice with PBS. After an additional 48 h of incubation, the original media were replaced with MEM (90 μL) and CCK-8 solution (10 μL), and the plates were incubated in a CO_2 incubator for 1–2 h. The absorbance at 450 nm was measured using a multimode microplate detection system. Untreated cells were used as controls, and cells treated with methanol were used as the blank.

10. Clonogenic assays

HT-1080 cells were cultured in 6-well plates, treated with **ABMI-COF**, **ABMBP-COF**, **siRNA@ABMI-COF**, and **siRNA@ABMBP-COF** (2.0 mL, 40 μg/mL, COF equiv.) for 4 h in a CO_2 incubator and washed twice with PBS. After approximately 7 days of incubation, the cells were fixed with paraformaldehyde (2.0 mL, 4 wt%) for 30 min and stained with fresh Giemsa staining solution for 1 h at room temperature. The plates were washed with water, air-dried, and photographed with a digital camera. Untreated wells were used as the control group, and wells without cells were used as the blank group.

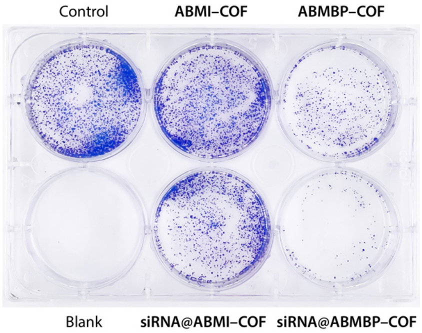

Fig. S6 *In vitro* clonogenic assays of HT-1080 cells treated with **ABMI-COF**, **ABMBP-COF**, **siRNA@ABMI-COF**, and **siRNA@ABMBP-COF** (40 μg/mL, COF equiv.).

11. Western blotting

HT-1080 cells were cultured in 6-well plates, treated with **ABMI-COF**, **ABMBP-COF**, **siRNA@ABMI-COF**, and **siRNA@ABMBP-COF** (2.0 mL, 40 μg/mL, COF equiv.) for 4 h in a CO_2 incubator and washed twice with PBS. After 48 h of additional culture, adherent cells were washed once in ice-cold PBS and lysed in ice-cold RIPA lysis and extraction buffer (Thermo Scientific, Cat# 89900) supplemented with a protease inhibitor cocktail (MedChemExpress, Cat# HY-K0010) and a phosphatase inhibitor cocktail (MedChemExpress, Cat# HY-K0022). The lysates were centrifuged at 13000 rpm at 4°C for 15 min, and the supernatant was collected. The protein concentration was quantified using a BCA protein assay kit (Thermo Fisher, Cat# 23227). Samples were prepared with SDS–PAGE sample loading buffer (Beyotime, Cat# P0015), and 20 μg of protein per sample was loaded and subjected to electrophoresis to separate the target proteins. After the proteins were transferred to a polyvinylidene difluoride membrane and blocked for 1 h using nonfat powdered milk (5 wt%), the membrane was incubated with primary antibodies at 4°C overnight. The primary antibodies used were rabbit anti-FSP1 (1:1000 dilution, Proteintech, Cat# 20886-1-AP), rabbit anti-SLC7A11 (1:1000 dilution, Cell Signaling, Cat# 12691), rabbit anti-ACSL4 (1:10000 dilution, Abcam, Cat# ab155282), rabbit anti-GPX4 (1:1000 dilution, Abcam, Cat# ab125066), and rabbit anti-β-tubulin (1:1000 dilution, HuaBio, Cat# ET1602-4). Subsequently, the membranes were washed 3 times for 10 min in Tris-buffered saline with 0.1 vol% Tween-20 and incubated with HRP-conjugated goat anti-rabbit IgG (H+L) secondary antibody (1:10000 dilution, Proteintech, Cat# SA00001-2) for 1 h at room temperature. The protein of interest was observed using a chemiluminescence detection kit (Vazyme, Cat# E412-01).

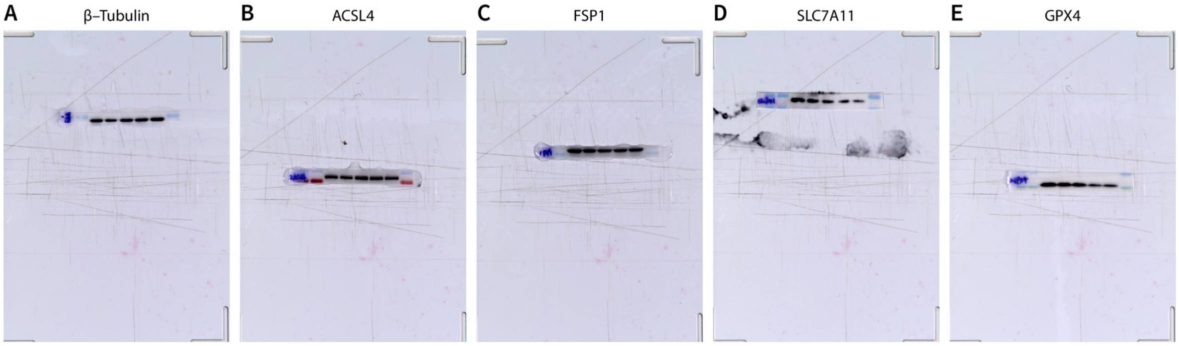

Fig. S7 Unedited original western blot images. (A) β-Tubulin. (B) ACSL4. (C) FSP1. (D) SLC7A11. (E) GPX4.

12. Enzymatic activity assays

HT-1080 cells were cultured in 6-well plates, treated with **ABMI-COF**, **ABMBP-COF**, **siRNA@ABMI-COF**, and **siRNA@ABMBP-COF** (1.0 mL, 40 μg/mL, COF equiv.) for 4 h in a CO_2 incubator and washed twice with PBS. After an additional 48 h of incubation, HK2 and GPX4 activity was analysed using commercially available kits. Untreated cells were used as the control group. HK2 and GPX4 activity was normalized to the total protein amount in the cell lysates from a parallel plate and are expressed as a percentage relative to the control group.

13. Intracellular GSH and malondialdehyde measurement

HT-1080 cells were cultured in 6-well plates, treated with **ABMI-COF**, **ABMBP-COF**, **siRNA@ABMI-COF**, and **siRNA@ABMBP-COF** (2.0 mL, 40 μg/mL, COF equiv.) for 4 h in a CO_2 incubator and washed twice with PBS. After an additional 48 h of incubation, GSH and malondialdehyde measurements were performed using commercially available kits. Untreated cells were used as the control group. GSH and malondialdehyde levels were normalized to the total protein amount in the cell lysates from a parallel plate.

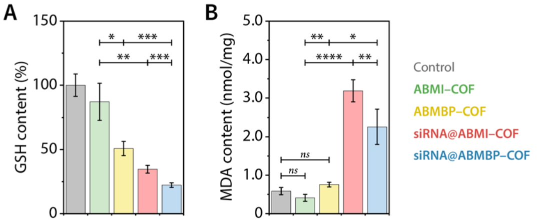

Fig. S8 Intracellular GSH (A) and malondialdehyde (B) levels in HT-1080 cells treated with **ABMI-COF**, **ABMBP-COF**, **siRNA@ABMI-COF**, and **siRNA@ABMBP-COF** (40 μg/mL, COF equiv.) The data are presented as the mean ± SD ($n = 5$) and were compared by Welch's ANOVA test followed by Dunnett's T3 multiple comparison test. *$p < 0.05$, **$p < 0.01$, ***$p < 0.001$, ****$p < 0.0001$, *ns*, no significance ($p > 0.05$).

14. Intracellular Fe^{2+} analysis

HT-1080 cells were cultured in 8-well chamber slides, treated with **ABMI-COF**, **ABMBP-COF**, **siRNA@ABMI-COF**, and **siRNA@ABMBP-COF** (200 μL, 100 μg/mL, COF equiv.) for 4 h in a CO_2 incubator and washed twice with PBS. Then, the cells were incubated with DFOM (200 μL, 50 μM) for 48 h. Subsequently, the cells were loaded with FerroOrange (200 μL, 20 μM) for 30 min in a CO_2 incubator. Laser scanning confocal fluorescence images were captured with the orange signal recorded at an excitation wavelength of 561 nm and an emission wavelength range of 570–620 nm. Untreated cells were used as the control group.

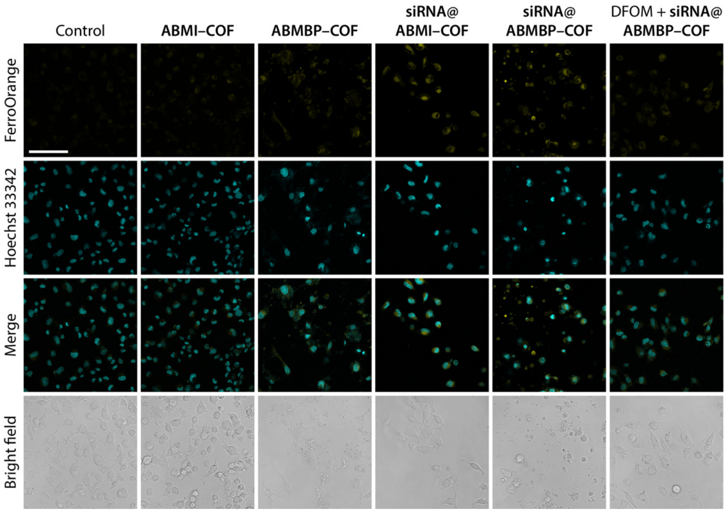

Fig. S9 Laser scanning confocal fluorescence microscopy images of HT-1080 cells treated with **ABMI-COF**, **ABMBP-COF**, **siRNA@ABMI-COF**, and **siRNA@ABMBP-COF** (100 μg/mL, COF equiv.) showing intracellular Fe^{2+} using FerroOrange (20 μM) as a fluorescence probe. Scale bar, 100 μm.

15. Intracellular ROS measurement

HT-1080 cells were treated with **ABMI-COF**, **ABMBP-COF**, **siRNA@ABMI-COF**, and **siRNA@ABMBP-COF** (200 μL, 40 μg/mL, COF equiv.) for 4 h in a CO_2 incubator and washed twice with PBS. After an additional 48 h of incubation, the cells were loaded with DCFH-DA (200 μL, 20 μM) for 30 min in a CO_2 incubator and then washed twice with PBS. Laser scanning confocal fluorescence images were captured with the green signal recorded at an excitation wavelength of 488 nm and an emission wavelength range of 500–540 nm. Untreated cells were used as the control group.

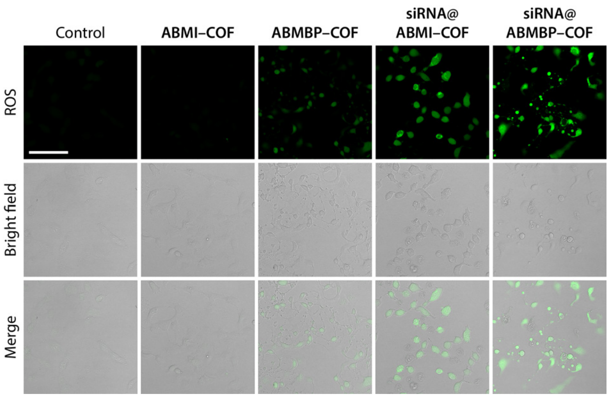

Fig. S10 Laser scanning confocal fluorescence microscopy images of HT-1080 cells treated with **ABMI-COF**, **ABMBP-COF**, **siRNA@ABMI-COF**, and **siRNA@ABMBP-COF** (40 µg/mL, COF equiv.) showing total intracellular ROS levels using DCFH-DA (20 µM) as a fluorescence probe. Scale bar, 100 µm.

16. Intracellular lipid peroxide measurement

HT-1080 cells were treated with **ABMI-COF**, **ABMBP-COF**, **siRNA@ABMI-COF**, and **siRNA@ABMBP-COF** (200 µL, 40 µg/mL, COF equiv.) for 4 h in a CO_2 incubator and washed twice with PBS. After an additional 48 h of incubation, the cells were loaded with C_{11}-BODIPY (200 µL, 2.0 µM) for 30 min in a CO_2 incubator and then washed twice with PBS. Laser scanning confocal fluorescence images were captured. The green signal of the oxidized C_{11}-BODIPY was recorded at an excitation wavelength of 488 nm and an emission wavelength range of 490–530 nm. The red signal of the reduced C_{11}-BODIPY was recorded at an excitation wavelength of 561 nm and an emission wavelength range of 570–610 nm. Untreated cells were used as the control group.

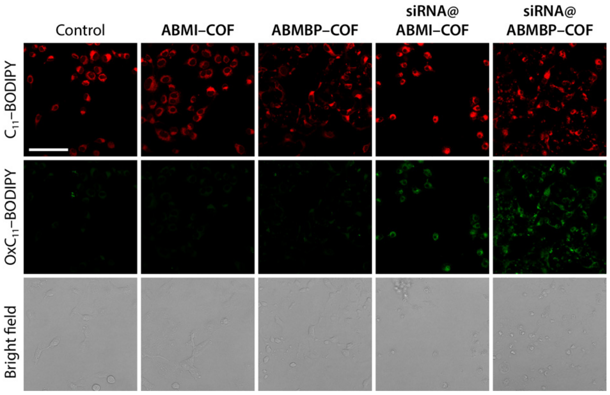

Fig. S11 Laser scanning confocal fluorescence microscopy images of HT-1080 cells treated with **ABMI-COF**, **ABMBP-COF**, **siRNA@ABMI-COF**, and **siRNA@ABMBP-COF** (40 µg/mL, COF equiv.) showing intracellular lipid peroxide using C_{11}-BODIPY (2.0 µM) as a fluorescence probe. Scale bar, 100 µm.

17. Mitochondrial membrane potential measurement

HT-1080 cells were treated with **ABMI-COF**, **ABMBP-COF**, **siRNA@ABMI-COF**, and **siRNA@ABMBP-COF** (200 µL, 40 µg/mL, COF equiv.) for 4 h in a CO_2 incubator and washed twice with PBS. After an additional 48 h of incubation, the cells were incubated with JC-1 (200 µL, 15 µM) for 10 min in a CO_2 incubator and washed twice with PBS. Laser scanning confocal fluorescence images were captured. The green signal of the JC-1 monomer was detected by excitation at 488 nm and emission at 530±15 nm. The red signal of *J*-aggregate JC-1 was detected by excitation at 561 nm and emission at 590±17 nm. Untreated cells were used as the control group.

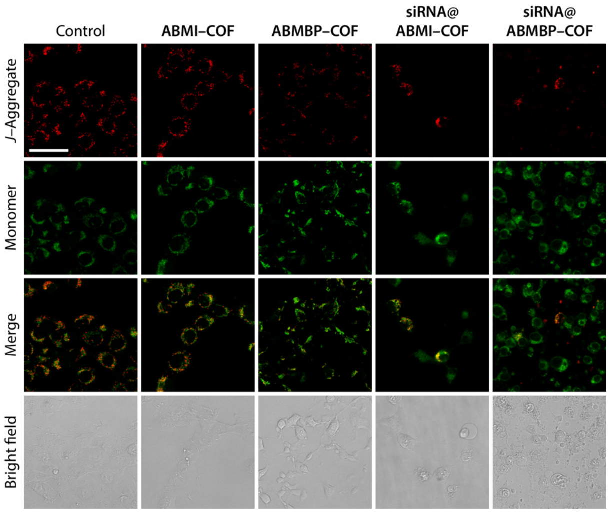

Fig. S12 Laser scanning confocal fluorescence microscopy images of HT-1080 cells treated with **ABMI-COF**, **ABMBP-COF**, **siRNA@ABMI-COF**, and **siRNA@ABMBP-COF** (40 µg/mL, COF equiv.) showing mitochondrial membrane potentials using JC-1 (15 µM) as a fluorescence probe. Scale bar, 50 µm.

18. Immunofluorescence imaging of active caspase 3

HT-1080 cells were treated with **ABMI-COF**, **ABMBP-COF**, **siRNA@ABMI-COF**, and **siRNA@ABMBP-COF** (200 µL, 40 µg/mL, COF equiv.) for 4 h in a CO_2 incubator and carefully washed with PBS. After an additional 48 h of incubation, the cells were fixed in ice-cold paraformaldehyde (400 µL, 4 wt%) for 20 min and washed twice with PBS. The cells were permeabilized with Triton X-100 (500 µL, 0.5 vol%) for 5 min and washed twice with PBS. The cells were incubated in PBS (500 µL) containing normal goat serum (10 vol%) and Tween-20 (0.1 vol%) for 1 h at room temperature to block nonspecific protein interactions. The cells were incubated with rabbit anti-active caspase 3 primary antibody (1:100 dilution, Thermo Fisher, Cat# MA5-32015) at 4°C overnight. The cells were washed three times with PBS and incubated with Alexa Fluor 488-conjugated goat anti-rabbit IgG (H+L) secondary antibody (1:500 dilution, Thermo Fisher, Cat# A-11008) for 1 h at room temperature. The cell nuclei were counterstained with propidium iodide (200 µL, 3.0 µM) for 30 min at room temperature and washed twice with PBS. Laser scanning confocal fluorescence images were captured. The green signal of Alexa Fluor 488 and the red signal of propidium iodide were recorded at an excitation wavelength of 488 nm and emission wavelength ranges of 500–540 nm and 590–620 nm, respectively. Untreated cells were used as the controls.

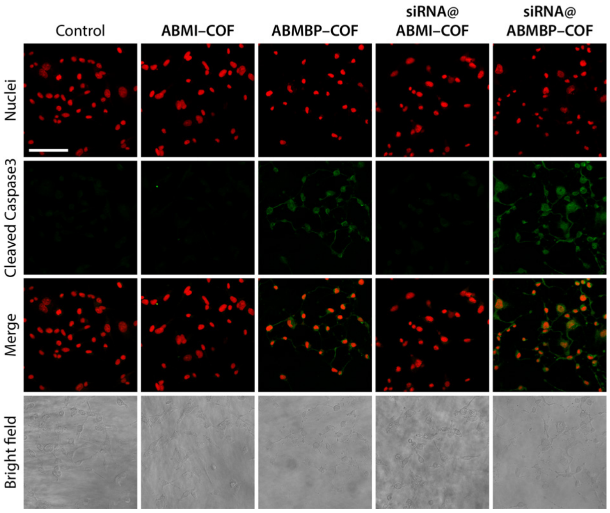

Fig. S13 Immunofluorescence images of cleaved caspase 3 in HT-1080 cells treated with **ABMI-COF**, **ABMBP-COF**, **siRNA@ABMI-COF**, and **siRNA@ABMBP-COF** (40 µg/mL, COF equiv.). Scale bar, 100 µm.

19. Cell death rescue experiments

HT-1080 cells were cultured in 96-well plates, treated with **siRNA@ABMBP-COF** (100 μL, 40 μg/mL, COF equiv.) for 4 h in a CO_2 incubator and washed twice with PBS. Different inhibitors or agonists, including sodium pyruvate (100 μL, 2.5 mM), glutathione ethyl ester (100 μL, 1.0 mM), *N*-acetyl-*L*-cysteine (100 μL, 1.0 mM), 2-mercaptoethanol (100 μL, 1.0 mM), ferrostatin-1 (100 μL, 1.0 μM), Z-VAD-FMK (100 μL, 50 μM), necrostatin-1 (100 μL, 0.5 μM), and 3-methyladenine (100 μL, 50 μM), were added to each well. After an additional 48 h of incubation, the cells were washed twice with PBS. Then, MEM (90 μL) and CCK-8 solution (10 μL) were added to each well, and the plate was incubated in a CO_2 incubator for 1 h. The absorbance at 450 nm was determined using a multimode microplate detection system. Untreated cells were used as controls, and cells treated with methanol were used as the blank.

20. Experimental animals

All animal procedures were reviewed and approved by the Ethics Committee of Shandong Normal University (Jinan, China; application number AEECSDNU2021009). All animal experiments complied with the relevant guidelines of the Chinese government and the regulations for the care and use of experimental animals.

BALB/c nude mice (♀) were purchased from Beijing Vital River Laboratory Animal Technology Co., Ltd. The nude mice were housed in a filter-topped facility (pathogen-free) with autoclaved food and water and were kept in a temperature-controlled room set to a 12:12 h light-dark cycle.

21. *In vivo* antitumor efficacy

HT-1080 cells (approximately 10^6 cells) suspended in HBSS (40 μL) were subcutaneously injected into the flanks of each nude mouse (aged 4 weeks, ♀) to establish the HT-1080 xenograft model. The length (L) and width (W) of the tumour were measured using digital callipers. The tumour volume (V) was calculated using the following formula: $V = 1/2 \times L \times W^2$. Nude mice that failed to develop tumours from the beginning of the experiment were excluded.

When the tumour size reached 100–150 mm^3, the nude mice were randomly divided into 6 groups. In groups 1–5, the mice were intratumorally injected with PBS, **ABMI-COF**, **ABMBP-COF**, **siRNA@ABMI-COF**, and **siRNA@ABMBP-COF** (40 μL, 0.8 mg/mL, COF equiv.), respectively. Group 6 was peritumorally injected with liproxstatin-1 (10 mg/kg) on days 0, 2, 4, 6, and 8, in addition to receiving the same treatment as group 5. The tumour size of each mouse was measured using a calliper, and body weight was measured using an electronic scale.

On the 4th day after nanomedicine injection, 4 mice in each group were randomly selected and euthanized. The harvested tumour tissues were washed with normal saline containing heparin (160 μg/mL), cut into small pieces (approximately 10 mg), and homogenized in Tris-HCl buffer (10 mM, pH=7.4) containing protease inhibitor cocktail (MedChemExpress, Cat# HY-K0010) and EDTA-2Na (0.1 mM) at 4°C. The tumour homogenates were centrifuged at 3500 rpm for 15 min at 4°C, and GSH levels, malondialdehyde levels, HK2 activity, and GPX4 activity in the supernatants were measured with assay kits according to the manufacturers' guidelines. In addition, the total protein level was measured in parallel using a BCA protein assay kit (Thermo Fisher, Cat# 23227). The data were normalized to total protein level and are expressed as an absolute value or a percentage value relative to the control group value.

The remaining mice were further reared. When the tumour size reached 15 mm in either dimension, the *in vivo* antitumor experiment was terminated, and all mice were euthanized. The tumour tissue and major organs were collected for H&E histological analysis, Ki67

immunohistochemical staining, and TUNEL immunofluorescence staining.

Furthermore, to evaluate the biocompatibility of the nanodrugs, nude mice (aged 8 weeks, ♀) were randomly divided into 5 groups and intravenously injected with PBS, **ABMI-COF**, **ABMBP-COF**, **siRNA@ABMI-COF**, and **siRNA@ABMBP-COF** (80 μL, 0.4 mg/mL, COF equiv.). The nude mice were housed for 7 days, and the blood of each mouse was collected from the eyes for blood biochemical testing.

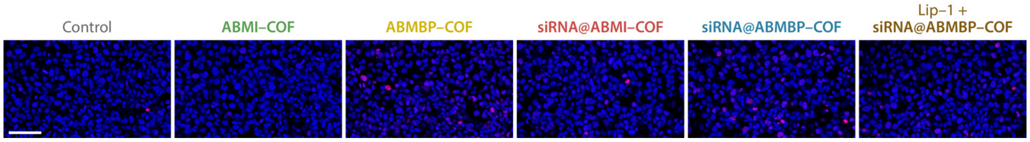

Fig. S14 TUNEL immunofluorescence staining of the obtained tumours. Scale bar, 100 μm.

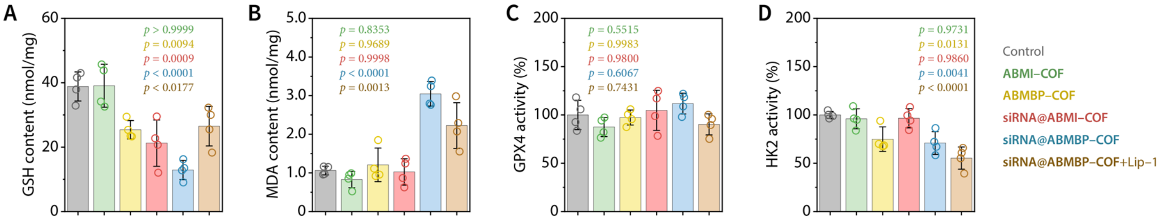

Fig. S15 Analysis of metabolite levels and enzymatic activity in tumours on day 4. (A) GSH levels. (B) Malondialdehyde levels. (C) GPX4 activity. (D) HK2 activity. The data are presented as the mean ± SD ($n = 4$) and were compared by one-way ANOVA followed by Dunnett's post-hoc test.

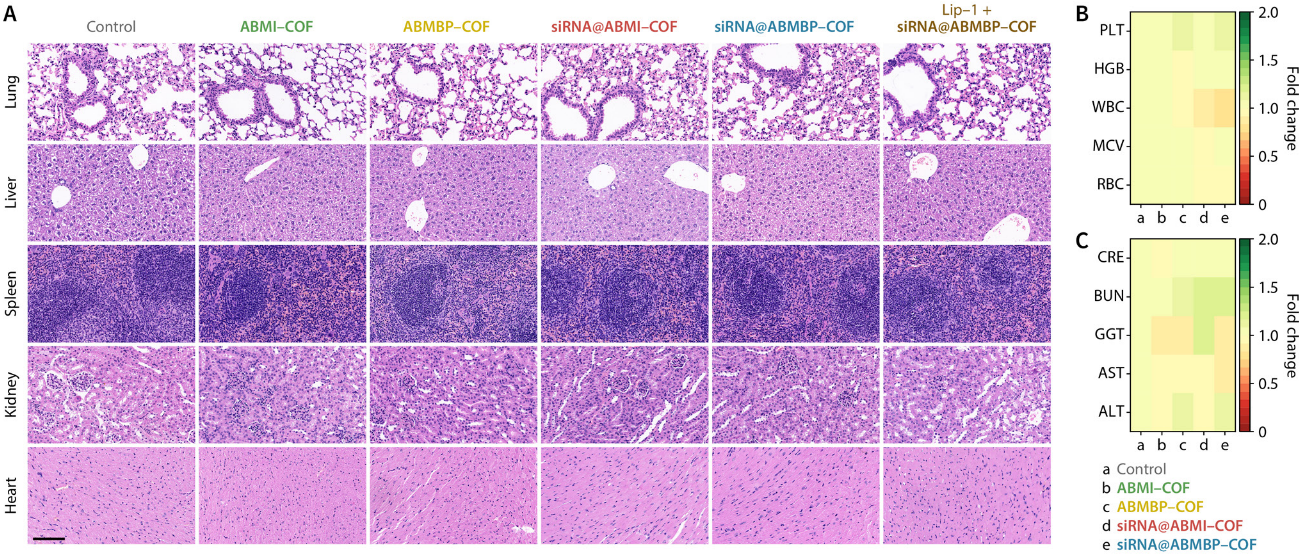

Fig. S16 Biosafety assessments. (A) H&E staining of the main organs collected at the end of the treatment. Scale bar, 100 μm. (B–C) Routine blood examination (B) and blood biochemical indices (C) of healthy nude mice that were intravenously injected with PBS, **ABMI-COF**, **ABMBP-COF**, **siRNA@ABMI-COF**, and **siRNA@ABMBP-COF** (80 μL, 0.4 mg/mL, COF equiv.). The data were normalized and are presented as the mean ($n = 5$) in heatmaps. PLT, platelet count; HGB, hemoglobin concentration; WBC, white blood cell count; MCV, mean corpuscular volume; RBC, red blood cell count; CRE, creatinine; BUN, blood urea nitrogen; GGT, γ-glutamyl transferase; AST, aspartate aminotransferase; ALT, alanine aminotransferase.
